# The Sickle Cell Disease Ontology: recent development and expansion of the universal sickle cell knowledge representation

**DOI:** 10.1093/database/baac014

**Published:** 2022-04-01

**Authors:** Gaston K Mazandu, Jade Hotchkiss, Victoria Nembaware, Ambroise Wonkam, Nicola Mulder

**Affiliations:** Department of Pathology, Division of Human Genetics, University of Cape Town, Health Sciences Campus, Anzio Rd, Observatory 7925, South Africa; Department of Pathology, Division of Human Genetics, University of Cape Town, Health Sciences Campus, Anzio Rd, Observatory 7925, South Africa; Department of Pathology, Division of Human Genetics, University of Cape Town, Health Sciences Campus, Anzio Rd, Observatory 7925, South Africa; Department of Pathology, Division of Human Genetics, University of Cape Town, Health Sciences Campus, Anzio Rd, Observatory 7925, South Africa; Department of Integrative Biomedical Sciences, Computational Biology Division, IDM, CIDRI-Africa WT Centre, University of Cape Town, Health Sciences Campus. Anzio Rd, Observatory 7925, South Africa

## Abstract

The Sickle Cell Disease (SCD) Ontology (SCDO, https://scdontology.h3abionet.org/) provides a comprehensive knowledge base of SCD management, systems and standardized human and machine-readable resources that unambiguously describe terminology and concepts about SCD for researchers, patients and clinicians. The SCDO was launched in 2016 and is continuously updated in quantity, as well as in quality, to effectively support the curation of SCD research, patient databasing and clinical informatics applications. SCD knowledge from the scientific literature is used to update existing SCDO terms and create new terms where necessary. Here, we report major updates to the SCDO, from December 2019 until April 2021, for promoting interoperability and facilitating SCD data harmonization, sharing and integration across different studies and for retrospective multi-site research collaborations. SCDO developers continue to collaborate with the SCD community, clinicians and researchers to improve specific ontology areas and expand standardized descriptions to conditions influencing SCD phenotypic expressions and clinical manifestations of the sickling process, e.g. thalassemias.

**Database URL**: https://scdontology.h3abionet.org/

## Introduction

The Sickle Cell Disease Ontology (SCDO) is the most comprehensive standardized domain knowledge portal, which unambiguously describes sickle cell disease (SCD) concepts and terminology (1, 2). In this ontology, existing SCD knowledge is hierarchically structured and represented in human and machine-readable formats, allowing researchers and clinicians to readily access standardized SCD‐related knowledge in a single location as well as enabling SCD information to be computationally processed and analyzed to support research (1, 3). The SCDO knowledge base is structured around the ‘Hemoglobinopathy’ class as a central aspect and links various classes as appropriate through relevant SCDO axioms (object properties), so that their relationships can be computationally inferred using logical reasoning (1). The SCDO structure has been meticulously designed over the course of 5 years (2) in a manner that takes into account the complexities of SCD. The SCDO is designed to be dynamic and is constantly being refined to keep its content up to date in order to effectively represent the most current knowledge of SCD. Updates and inclusions are made when there are new discoveries in the SCD research and clinical practice fields, for example, technological advances for diagnostics and therapeutics (4, 5). The core ontology development team is composed of experts in SCD and knowledge representation who engage curators to capture available SCD knowledge and to curate the ever-increasing amount of information in the literature.

The SCDO provides a global framework to address semantic challenges related to SCD data analytics. This framework aims to contribute to the creation of a healthcare system that supports disease management and research by providing an SCD clinical data dictionary (6) for mapping standardized metadata consistently. Here, we present important updates to the 2019 release (1). The SCDO currently contains 2072 concepts, of which 1950 are non-deprecated, covering various aspects of SCD, including phenotypes, diagnostics, therapeutics, disease modifiers, modes of inheritance, SCD-related complications, genetic phenomena and gene products, as well as other environmental and clinical research aspects (personal attributes, quality of life and quality of care for patients and research). The SCDO has 61 relations (object properties) for linking concepts. Definitions are provided for almost all SCDO concepts, and different ontology annotations have been updated to comply with the Open Biomedical Ontology (OBO) Foundry guiding principles (7). Concept Internationalized Resource Identifiers (IRIs) for terms have been updated to the standard OBO prefix provided by the OBO Foundry for the SCDO: http://purl.obolibrary.org/obo/scdo. This ensures interoperability with other OBO Foundry ontologies. Most notably, new classes have been included: mostly classes representing International Classification of Diseases, Tenth Revision, Clinical Modification (ICD-10 CM) Diagnosis Codes (8) relevant to SCD and a ‘Data Standards’ class, with numerous subclasses representing SCD data elements (DEs). With these recent changes, the SCDO is better set to ease SCD data harmonization, standardization, interoperability and integration tasks, thus enabling meta-analysis in clinical research, such as that to be carried out by the SickleInAfrica consortium (9). This new SCDO version is further expected to support digital SCD diagnostic and therapeutic processes (10) as well as to facilitate integration with electronic health records (EHRs), an ongoing challenge (10, 11) that has limited the use of EHR data in translational SCD research (11).

## Methods

### Expert curation and annotation quality control

The SCDO has a dedicated curation team assuring the quality and accuracy of the information contained in this ontology, and keeping it updated as SCD knowledge evolves. The updating process is iterative and collaborative, involving experts in the field to ensure that the SCDO consistently represents current SCD knowledge. When significant changes are to be implemented by curators, e.g. the addition of new top-level (close to the root) classes or new object property associations or when curators are uncertain of technicalities, changes or questions are discussed in detail with relevant SCDO working group components before implementing changes/edits into the WebProtege project (12, 13). Note that the SCDO working group has six components: phenotype, diagnostics, therapeutics, quality of life and care, disease modifiers and data standards (1, 3). The WebProtege tool, which provides a distributed ontology content management system, is used to enable domain experts, ontology curators and developers to share and update information, and easily visualize the ontology classes and structure. ROBOT (14), an open source tool for automating ontology development workflows and tasks, is used to compile the complete ontology release files, including Web Ontology Language (OWL) and OBO formats.

### SCDO interoperability

Complying with OBO Foundry guiding principles, curators cross-reference terms included from other ontologies. In this updated version, the cross-references are taken into account when the ontology file is compiled for a new release, replacing SCDO IRIs of terms used from external ontologies (those for which the ‘existence in other ontologies’ annotation property indicates ‘Sufficient’) with their IRIs from their source ontology. This allows SCDO terms from external ontologies to be annotated with additional information unique to the SCDO while still promoting interoperability with other ontologies and datasets. As an example, ‘Impaired Renal Concentrating Ability’ is reused from the HPO under the identifier HPO:0004727. In the SCDO, this term has additional annotations such as the exact synonym, ‘UCD’, and the age of onset, ‘Notable within the first decade of life.’

## Results

### SCDO resource content and new developments

The updated SCDO knowledge base contains 2072 terms, which include the ‘deprecated terms’ class containing 122 subclasses that are obsolete. In comparison to the previous release, there are 476 new concepts (1474 non-deprecated terms in 2019 vs 1950 in 2021). Most of the new terms are within the new ‘Data Standards’ upper-level class or are classes for new ICD-10 CM Diagnosis Codes relevant to SCD.

**Figure 1.**
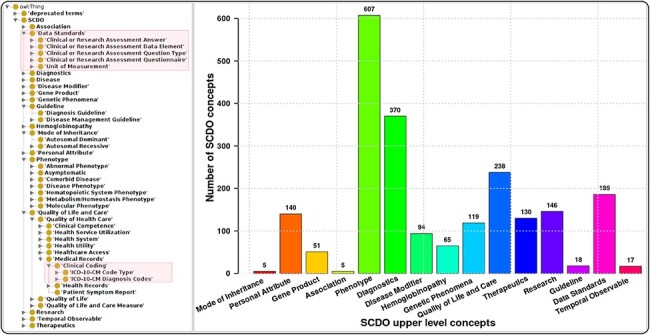
The SCDO tree view showing the hierarchical representation of knowledge on SCD with two main new concepts highlighted in the boxes on the left, and a bar graph showing the number of concepts under the different upper-level terms.

**Figure 2.**
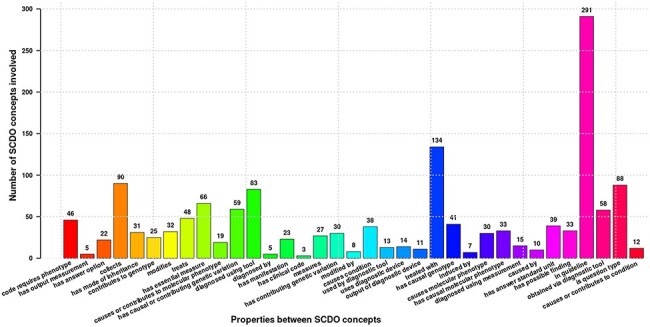
Different properties defined between SCDO concepts mapping the current SCD knowledge. Numbers at the top of bars represent the frequency of occurrence of the association in the ontology.

**Figure 3.**
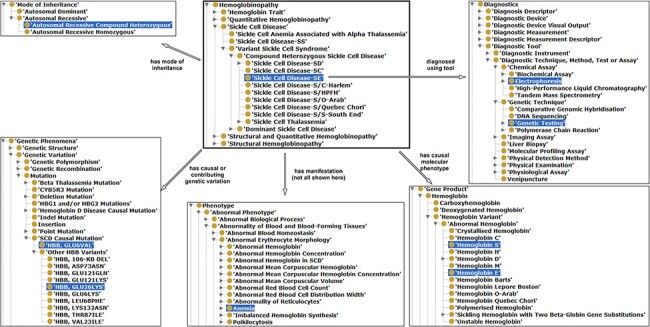
Illustration of properties between hemoglobinopathy concepts as an approach to integrating existing hemoglobinopathy knowledge. The ‘Sickle Cell Disease-SE’ class is the reference or the object, a subclass of hemoglobinopathy having some properties (annotation in different arrows) related with other classes (subjects) highlighted in blue.

The SCDO captures multiple aspects of SCD with a central ‘Hemoglobinopathy’ class. This class connects to 15 other high-level SCD concepts related to disease aspects, such as diagnostics, therapeutics, modifiers and phenotypes. Other connected aspects pertinent to SCD research, clinical practice, and patient outcomes and care include personal attributes (including ethnolinguistic groups), quality of life and quality of care, and guidelines to understand phenotypic manifestations and inform clinical management. The distribution of concepts within high-level concepts and the hierarchical ontology view are shown in Figure 1. These concepts are linked by 3847 relationships of which 2358 are topological (is_a) and others represent object properties defined between different SCDO concepts. The distribution of their occurrence is shown in Figure 2 with specific illustrations provided in Figure 3. It is important to note that the updates reflect two distinct processes: addition of annotations based on new evidence, and obsoleting of annotations that have been completely revised based on new information. An increase in the number of obsolete annotations is expected due to increased annotation quality assurance efforts, described in more detail later.

### SCDO new release highlights

**Figure 4.**
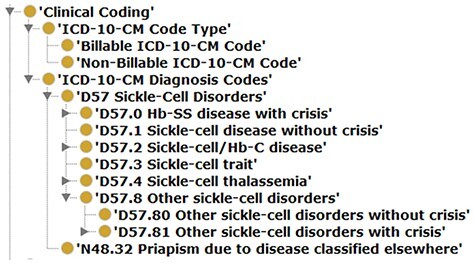
Illustrating a case of an updated SCDO concept—The ‘Clinical Coding’ class.

The SCDO (https://scdontology.h3abionet.org/) currently represents the largest knowledge base for SCD and other hemoglobinopathies. As highlighted in the previous release (1), existing related biomedical ontologies, such as the Human Phenotype Ontology (HPO) (15, 16), the Human Disease Ontology (DO) (17) and Monarch Disease Ontology (MonDO) (18) do not capture all SCD-specific concepts and therefore cannot serve as a comprehensive source of SCD knowledge. As for the previous version, the new SCDO version released on 15 April 2021 provides a standardized and structured vocabulary system describing SCD concepts consistently for research and clinical applications. The following are key features of the current release:

1. Compliance with OBO Foundry guiding principles: IRIs were updated to the standard OBO prefix (i.e. http://purl.obolibrary.org/obo/) and, where possible, SCDO IRIs were replaced by those from existing ontologies, thus fostering ontology interoperability. There are 1386 terms (71%) specific to the SCDO with stable machine-readable SCDO identifiers and human-readable labels, and 686 terms (29%) from other ontologies.
2. Simple Standard for Sharing Ontology Mappings (SSSOM) files accompany this release. The file ‘scdo-sssom-xrefs.tsv’ (https://github.com/scdodev/scdo-ontology/blob/master/src/ontology/mappings/scdo-sssom-xrefs.tsv) contains standard metadata elements to describe mappings between SCDO terms and terms in external ontologies. The file ‘scdo-sssom-xrefs-rename.tsv’ (https://github.com/scdodev/scdo-ontology/blob/master/src/ontology/mappings/scdo-sssom-xrefs-rename.tsv) lists the identifiers of SCDO terms that have their IRIs replaced by those of their cross-referenced terms from external ontologies, alongside the identifiers of these cross-referenced terms.
3. Ontology annotations have been updated and definitions or descriptions have been provided for almost all SCDO terms.
4. Inclusion of several new concepts (see Figure 1), such as new patient and research participants’ consent-related terms, enabling the SCDO to support the SCD research data collection process.
5. Inclusion of new classes, most notably the ‘Data Standards’ class and subclasses of the ‘Clinical Coding’ class, including SCD-related ICD-10 CM Diagnosis Codes (Figure 4) and an **‘**ICD-10-CM Code Types’ class with subclasses ‘Billable ICD-10-CM Code’ and ‘Non-Billable ICD-10-CM Code’. The addition of these new classes is intended to facilitate the mapping of SCD datasets to functional knowledge to enable the subsequent SCD research translation into clinical applications and policy guidelines, as well as to provide a computational representation of diagnostics in clinical applications.
6. Inclusion of new object properties (see Figure 2) for linking-related concepts, to enable inference of relationships between concepts computationally using logical reasoning.
7. Notably, new object properties and annotation properties were added in order to apply an ontology design pattern (ODP) that is under development in order to map SCD DEs to SCDO terms in a complex and useful way. This ODP also involves linking ICD-10 CM diagnosis code to phenotypes that are linked to DEs via object properties such as ‘code requires phenotype’.

### Data availability

The SCDO is freely available under the GNU General Public License (GPL: https://www.gnu.org/licenses/gpl-3.0.en.html) and accessible from the SCDO website at https://scdontology.h3abionet.org/. It is copyrighted to maintain the quality and integrity of the term vocabulary, meaning that any modification to the SCDO can only be done by SCDO developers and curators. The SCDO can also be accessed through several platforms, including:

- The European Bioinformatics Institute Ontology Look-up Service (19) at https://www.ebi.ac.uk/ols/ontologies/scdo
- The National Center for Biomedical Ontology (NCBO) Bioportal (20) at https://bioportal.bioontology.org/ontologies/SCDO
- FAIRsharing (21) at https://fairsharing.org/FAIRsharing.kkaq6pw.

Links to other SCDO-related resources are listed below:

- Ontology Development Kit: https://github.com/scdodev/scdo-ontology
- Standard Operating Procedures (SOPs): https://www.sickleinafrica.org/sops_booklet
- The Global SCD Registry Portal: https://www.sickleinafrica.org/registries-list

## Discussion

### SCDO-based data standardization

The SCDO is continually evolving to meet challenges related to large-scale phenotype and clinical datasets collected using current advanced clinical data management systems, such as the Research Electronic Data Capture (REDCap) platform (22). These systems have enabled the development of detailed case report forms (CRFs), resulting in big datasets with large numbers of variables from different locations, generally using different variable names, labels and codes, limiting direct prospective or retrospective comparisons.

The SickleInAfrica consortium (9) is the largest pan-African SCD research network to date, which has built a multinational registry containing clinical data of over 10 000 SCD patients in over 27 data collection instruments. SickleInAfrica core data elements, constituting the minimal set of data elements determined by the SickleInAfrica consortium to be essential for an SCD patient registry, have been mapped to the SCDO. These mappings use a complex ODP (not a simple one-to-one mapping) that involves subclasses of the new ‘Data Standards’ class (including terms that represent SickleInAfrica DEs, as well as terms that capture information about them, such as the question type and unit of measurement) and mappings of these to other relevant SCDO terms A more in-depth description of the ODP is beyond the scope of this paper and will be the subject of another paper planned for publication later this year.

This updated SCDO currently enables the implementation of an ontology-driven REDCap database by mapping different variables collected to SCDO concepts and incorporating an ontology-variable map in the database back-end. The inclusion of an SCDO terminology layer in the SCD REDCap database system provides online help during data collection and exploration processes and should contribute to the improvement of the data quality. This standardizes the representation of all existing SCD metadata, promotes metadata reuse and adherence to the FAIR (Findable, Accessible, Interoperable and Reusable) principles (23), ensuring that these metadata are both human-readable and computationally accessible. Finally, the SCDO is set to be an essential resource for designing ontology-based CRFs to collect biomedical data for potential prospective cohort studies, capturing demographics, anthropometrics, medical history, lifestyle and phenotypes.

### Moving to multilingual SCDO and adapting annotation terminology

One critical challenge is to introduce the SCDO into the dynamic clinical setting and indigenous environment. Considering the social stigma related to most rare diseases, including SCD, it is critical to consider challenges and potential solutions in ethical considerations and patient relevance to facilitate progress in the field and to potentially assist in the SCD public and clinical management. Therefore, communication is essential and the SCDO aims to produce translated versions of the ontology into languages broadly spoken in many of the SCD-burdened African countries, such as French and Portuguese, as well as a layperson’s version for non-experts, including patients, to increase the uptake within and outside the SCD community. There are plans to translate the layperson version of the SCDO into African languages such as Swahili. A French version is planned to be released in early 2022 for this current version of the SCDO. In this version, all labels and definitions of non-deprecated terms have been translated, first using auto-translation software which was followed up with manual reviewing by French- and English-speaking SCD experts. There is currently an ongoing discussion within the OBO Foundry community focusing on what the best protocol would be for managing ontology translations. In the context of SCDO, we are suggesting the use of comprehensive standard language tags for managing translated ontology files, moving a step forward to the implementation of a single file multilingual ontology for the next release.

### Community engagement and training

The SCDO strives to promote global SCD research collaboration, fostering a more participatory and inclusive knowledge representation system design to ensure global resource-wide adherence. From the start in 2016, the training has always been a critical component to make the best use of this ontology. For example, the ontology core content was built and consolidated through different workshops, engaging with different communities, including experts and researchers from different continents (1, 3), and most importantly, with SCD patient groups from Cape Town (South Africa), Dar-es-Salaam (Tanzania), Kumasi (Ghana) and Abuja (Nigeria). In the third workshop in 2018 (1), the structure of the ontology was refined to support basic and translational research and the derived SCDO scheme was used to design another disease-specific ontology, the Hearing Impairment Ontology (24). The most recent workshop was held in December 2019, just before the Coronavirus disease (COVID-19) pandemic outbreak, and aimed at harnessing the SCDO to harmonize data elements from retrospective multi-site studies, contributing to the SickleInAfrica cohort collaborative research.

To keep accessing the SCDO global user-base under the continued COVID-19 pandemic threat, we have started moving away from face-to-face style workshops and training meetings toward use of distance or online meetings. We created an infographic video that describes what the SCDO is; its purpose, content and applicability; and how basic researchers might make use of it (https://scdontology.h3abionet.org/). To reach new communities, the video was widely advertised through social media forums, such as Twitter (@SickleInAfrica) or established mailing lists. Finally, we really value the interaction with and feedback and updates from our user community via the contact link on the SCDO website or direct emails to helpdesk@sickleinafrica.org.

## Conclusion

The SCDO aims to play a key role in SCD knowledge organization and representation, data collection and standardization, to create a reference framework for multiscale SCD biomedical data integration and analysis. As mentioned previously, the SCDO addresses the issue of unifying clinical research data from diverse sources and paves the way toward the integration of clinical data into electronic medical records. This may facilitate the development of effective health informatics tools to potentially assist in basic research, clinical and translational applications, including guiding diagnostics, gene-disease-phenotype-drug association prediction and data analytics, thus assisting in realizing optimal SCD therapy, biomedicine and precision medicine.

## Data Availability

The data underlying this article are available in [Ontology IRI] at [http://purl.obolibrary.org/obo/scdo.owl] and in [SCDO website] at [https://scdontology.h3abionet.org/].

## Appendix

**Table:** Sickle Cell Disease Ontology Working Group

Name	Surname	Affiliation	Preferred e-mail address
Marsha	Treadwell	University of California San Francisco, USA	marsha.treadwell@ucsf.edu
Baba	Inusa	Paediatric Haematology, Evelina London Children’s Hospital, Guy’s and St Thomas NHS Foundation Trust, London SE1 7EH	baba.inusa@kcl.ac.uk
Vimal	Derebail	University North Carolina at Chapel Hill, Chapel Hill, NC, USA	vimal_derebail@med.unc.edu
Obiageli	Nnodu	Centre of Excellence for Sickle Cell Disease Research and Training (CESRTA), University of Abuja of Abuja, Abuja, Nigeria	oennodu@gmail.com
Solomon Fiifi	Ofori-Acquah	West African Genetic Medicine Centre (WAGMC), College of Health Sciences, University of Ghana, Accra, Ghana. Centre for Translational and International Hematology, Vascular Medicine Institute, University of Pittsburgh, Pittsburgh, USA	sfo2@pitt.edu
Kofi A.	Anie	London North West University Healthcare NHS Trust and Imperial College London, UK	kofi.anie@nhs.net
Andrew D	Campbell	Children’s National Hospital, George Washington University School of Medicine and Health Sciences, Washington, DC, USA	acampbell@childrensnational.org
Kais	Ghedira	Laboratory of Bioinformatics, Biomathematics and Biostatistics, Institut Pasteur de Tunis, University of Tunis El Manar, Tunisia	kais.ghedira@pasteur.tn
Bamidele	Tayo	Department of Public Health Sciences, Loyola University Chicago, Maywood, IL, USA	btayo@luc.edu
Neil	Hanchard	Department of Molecular and Human Genetics, Baylor College of Medicine, Houston, TX, USA	hanchard@bcm.edu
Sarah	Kiguli	Department of Paediatrics and Child Health, Makerere University, Uganda	skwalube@yahoo.co.uk
Charmaine	Royal	Duke University, Durham, NC, USA	charmaine.royal@duke.edu
Deogratias	Munube	Department of Paediatrics and Child Health, Mulago National Referral Hospital/Makerere University, Kampala, Uganda	deomunube@gmail.com
Munung	Nchangwi Syntia	Division of Human Genetics, Department of Pathology, Faculty of Health Sciences, University of Cape Town, Cape Town, South Africa	nchangwisyntia@yahoo.com
Mario	Jonas	Division of Human Genetics, Department of Pathology, Faculty of Health Sciences, University of Cape Town, Cape Town, South Africa	Mario.jonas@uct.ac.za
Sangeda	Raphael Z	Department of Pharmaceutical Microbiology, Muhimbili University of Health and Allied Sciences, Tanzania	sangeda@gmail.com
Makani	Julie	Sickle Cell Programme, Department of Haematology and Blood Transfusion, Muhimbili University of Health and Allied Sciences, Tanzania	jmakani@blood.ac.tz
